# Image of the Month: Decision-Making in Surgery for Late Onset Hirschsprung Disease

**DOI:** 10.1055/s-0040-1721049

**Published:** 2020-12-03

**Authors:** Anisha Apte, Elise McKenna, Marc A. Levitt

**Affiliations:** 1 Department of Surgery, The George Washington University School of Medicine and Health Sciences, Washington, District of Columbia, United States; 2 Department of General and Thoracic Surgery, Children's National Medical Center, Washington, District of Columbia, United States; 3 Division of Colorectal and Pelvic Reconstruction Surgery, Children's National Medical Center, Washington, District of Columbia, United States

**Keywords:** Hirschsprung disease, chronic distension, colonic dysmotility, chronic constipation, pull-through

## Abstract

We present a case of a 14-year-old boy with chronic distension, poor growth, and chronic constipation. He undergoes anorectal manometry and rectal biopsy, confirming the diagnosis of Hirschsprung disease (HD). The case is presented with a key image and associated questions to prompt discussion on strategies for management and treatment of HD in late-diagnosed children.

## Case Report

**Fig. 1:**
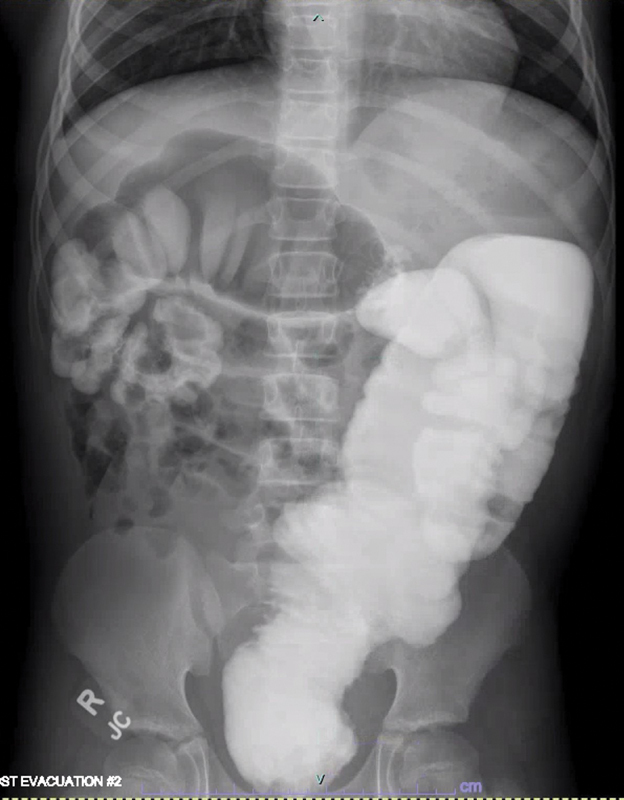
Contrast enema study.

A 14-year-old boy with chronic abdominal distension, poor growth (less than 1 percentile for weight), and chronic constipation presents to your clinic. He reports a history of passing hard stools every 2 to 3 days without the need for laxatives or an enema. At least once a week, he experiences fecal soiling. He has never had any episodes of enterocolitis. On abdominal exam, he has palpable stool burden. You are suspicious of Hirschsprung disease and obtain an awake anorectal manometry which shows an absent rectoanal inhibitory reflex. A rectal biopsy shows no ganglion cells and hypertrophic nerves, confirming the diagnosis of Hirschsprung disease (HD). His contrast enema study is demonstrated in Fig. 1 .

## Discussion

Late diagnosed HD (in children over the age of 2 years) presents differently than in the newborn period and warrants a different approach for surgical planning and management. Constipation is the number one presenting symptom in this group of patients 1 and they are more likely to have shorter segment disease with a transition zone in the rectosigmoid, and often in the rectum itself. These patients are less likely to present with enterocolitis, perhaps due to adaptive changes by the colon reacting to chronic distension, including hypertrophy and dilation. 1 Or such patients might have a different mucosal immunity, making bacterial translocation less likely.

In deciding on a surgical approach for patients with HD, the age of the patient should be factored into the decision-making, in addition to location of transition zone, extent of colonic dilation, as well the patient's nutritional status. 2 3 In the case presented, the contrast enema demonstrates a low transition zone at the rectosigmoid junction with dilation of the sigmoid colon extending to the descending colon. His history also suggests malnutrition and failure to thrive, not uncommon in HD, but much rarer in functional constipation and colonic motility disorders. We will discuss how each of these factors influence the decision to proceed in this case with a diverting ileostomy with colonic biopsies and future pull-through.

**Fig. 2:**
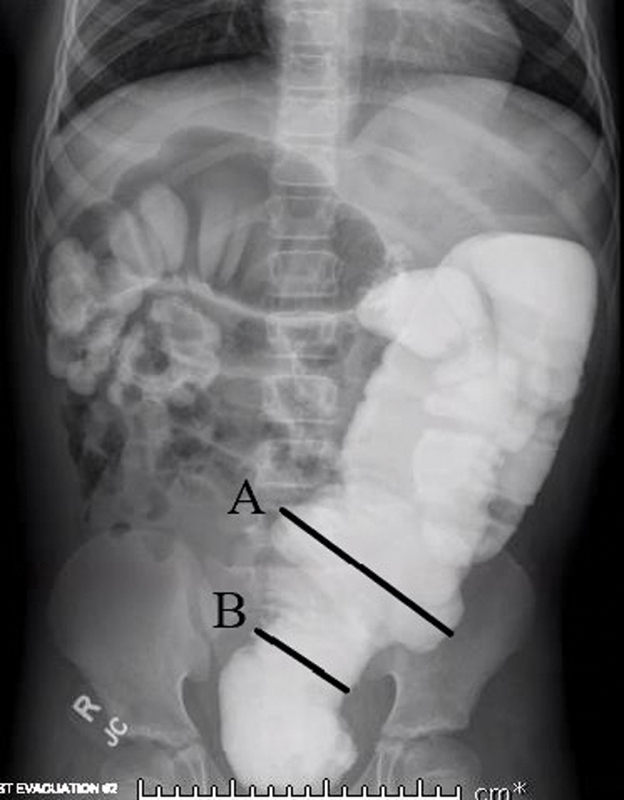
Contrast enema study with demonstration of transition zone at rectosigmoid junction. ( **A** ) Sigmoid colon. ( **B** ) Rectosigmoid junction and transition zone.

The location of the transition zone guides whether the patient can undergo a transanal only approach or whether laparoscopic assistance will be needed. A low transition zone, in the midsigmoid colon or lower, is a unique opportunity for a transanal only approach, as colonic mobilization is usually not required to create a tension-free anastomosis. 2 However, a laparoscopic mobilization of the distal rectum and sigmoid prevents stretching of the anal canal during the rectal dissection. Such overstretching of the sphincters is one of the main causes of postoperative morbidity and must be avoided. 4 If the transition zone is in the proximal sigmoid or above, mobilization of lateral and retroperitoneal attachments may be needed to ensure adequate length. 2 The contrast enema is very helpful in identifying the likely transition zone as the surgeon plans for surgery ( Fig. 2 ); however, the level must be confirmed by colonic biopsy. In this patient, a primary pull-through would not be appropriate despite a low transition zone, given the presence of significant colonic dilation and malnutrition.

Dilated bowel presents a significant technical challenges with visualization during laparoscopy, which may lead to nearby structural injuries. 2 A transanal dissection has been noted to lead to excessive stretching of the anal sphincter, which can lead to incontinence, 5 and a dilated colon anastomosed to the canal with a significant size discrepancy is a risk factor for an anastomotic dehiscence. Malnutrition is a known risk factor for anastomotic leak.

Intestinal diversion and decompression are recommended to address these issues. A leveling colostomy in most clinical settings is preferred, as an ileostomy does put the patient at risk for dehydration. 5 During enterostomy creation, colonic biopsies assessed on permanent section can more reliably confirm the location of the transition zone compared with frozen section analysis. While regular colonic irrigations are another option for decompressing dilated colon, they commonly do not provide adequate decompression in older children, who also may be less likely to tolerate regular irrigations. 5

## Conclusion

In summary, while one-stage pull-through procedures are convenient and desirable due to reduced morbidities of stoma formation, these may be limited to children with low transition zones and minimal to no proximal colonic dilation. In the rare circumstance of a late-diagnosed child with HD with proximal colonic dilation and/or malnutrition, a decompressive ostomy as a first step, and a plan for a future pull-through is a safer option to help prevent complications such as anastomotic leak, incontinence, obstruction, and need for a repeat pull-through. In an older child, the presentation of HD is often more subtle, with constipation, failure to thrive, and chronic distension being the dominant symptoms. It is rare that such patients have experienced enterocolitis. Why this case is unclear, and points to the broad spectrum of phenotypic expression of the disease.

## Notes

New Insights and the Importance for the Pediatric Surgeon In an older child, the presentation of Hirschsprung disease is often more subtle, with constipation, failure to thrive, and chronic distension being the dominant symptoms. For the late-diagnosed Hirschsprung disease patient with proximal colonic dilation and/or malnutrition, a decompressive ostomy as a first step, and a plan for a future pull-through is a safer option to help prevent complications such as anastomotic leak, incontinence, obstruction, and need for a repeat pull-through.
